# Molecular characterization and mapping of glucose-6-phosphate dehydrogenase (G6PD) mutations in the Greater Mekong Subregion

**DOI:** 10.1186/s12936-019-2652-y

**Published:** 2019-01-23

**Authors:** Germana Bancone, Didier Menard, Nimol Khim, Saorin Kim, Lydie Canier, Chea Nguong, Koukeo Phommasone, Mayfong Mayxay, Sabine Dittrich, Malavanh Vongsouvath, Nadine Fievet, Jean-Yves Le Hesran, Valerie Briand, Sommay Keomany, Paul N. Newton, Gornpan Gorsawun, Kaelan Tardy, Cindy S. Chu, Orpreeya Rattanapalroj, Le Thanh Dong, Huynh Hong Quang, Nguyen Tam-Uyen, Nguyen Thuy-Nhien, Tran Tinh Hien, Michael Kalnoky, Francois Nosten

**Affiliations:** 1 Shoklo Malaria Research Unit, Mahidol–Oxford Tropical Medicine Research Unit, Faculty of Tropical Medicine, Mahidol University, Mae Sot, Thailand; 2 Centre for Tropical Medicine & Global Health, Nuffield Department of Medicine, University of Oxford, Oxford, UK; 3 Malaria Molecular Epidemiology Unit, Institut Pasteur du Cambodge, Phnom Penh, Cambodia; 4 Malaria Genetics and Resistance Group, Institut Pasteur, Paris, France; 5 National Centre for Parasitology, Entomology and Malaria Control (CNM), Phnom Penh, Cambodia; 6 Microbiology Laboratory, Lao-Oxford-Mahosot Hospital-Wellcome Trust Research Unit (LOMWRU), Vientiane, Lao People’s Democratic Republic; 7 Institute of Research and Education Development, University of Health Sciences, Ministry of Health, Vientiane, Lao People’s Democratic Republic; 8 Foundation for Innovative New Diagnostics (FIND), Geneva, Switzerland; 9 UMR216-MERIT, French National Research Institute for Sustainable Development (IRD), Paris-5 University, Sorbonne Paris Cité, Paris, France; 10 Salavan Provincial Hospital, Salavan, Lao People’s Democratic Republic; 11 Bureau of Vector Born Diseases, Bangkok, Thailand; 12 Institute of Malariology, Parasitology and Entomology – Ho Chi Minh City (IMPE-HCM), Ho Chi Minh City, Vietnam; 13 Institute of Malariology, Parasitology and Entomology – Quy Nhon (IMPE-QN), Quy Nhon, Vietnam; 14 Oxford University Clinical Research Unit, Wellcome Trust Asia Program, in partnership with Hospital For Tropical Diseases (HTD), Ho Chi Minh City, Vietnam; 15 Diagnostics Program, PATH, Seattle, USA

## Abstract

**Background:**

*Plasmodium vivax* malaria elimination can only be achieved by the deployment of 8-aminoquinolines (primaquine and tafenoquine) in combination with ACT to kill both blood and liver-stage parasites. However, primaquine and the other 8-aminoquinolines cause dose-dependent haemolysis in subjects with G6PD deficiency, an X-linked disorder of red blood cells that is very common in populations living in tropical and subtropical areas. In order to inform safer use of 8-aminoquinolines in the Greater Mekong Subregion, a multi-centre study was carried out to assess the prevalence of G6PD deficiency and to identify the main G6PD variants in samples collected in Cambodia, Lao PDR, Myanmar, Thailand and Vietnam.

**Methods:**

Blood samples were collected in the five countries during National Malaria Surveys or during Population Surveys. During Population Surveys samples were characterized for G6PD phenotype using the Fluorescent Spot Test. Samples were then genotyped for a panel of G6PD mutations.

**Results:**

G6PD deficiency was found to be common in the region with an overall mean prevalence of deficient or mutated hemizygous males of 14.0%, ranging from a mean 7.3% in Thailand, 8.1% in Lao PDR, 8.9% in Vietnam, 15.8% in Myanmar and 18.8% in Cambodia. Mahidol and Viangchan mutations were the most common and widespread variants found among the nine investigated.

**Conclusions:**

Owing to the high prevalence of G6PD deficiency in the Greater Mekong Subregion, strategies for vivax malaria elimination should include point-of-care G6PD testing (both qualitative and quantitative) to allow safe and wide treatment with 8-aminoquinolines.

**Electronic supplementary material:**

The online version of this article (10.1186/s12936-019-2652-y) contains supplementary material, which is available to authorized users.

## Background

In South-East Asia, the incidence of malaria (mainly *Plasmodium falciparum*) has been significantly reduced in the past decade thanks to the implementation of integrated strategies combining vector control, early diagnosis and treatment with ACT, and the fight against falsified and substandard anti-malarials. However, the emergence of *P. falciparum* resistance to artemisinin and partner drugs (mefloquine, piperaquine) is threatening this progress [1–4]. This has led to the deployment of further interventions including mass drug administration to accelerate *P. falciparum* elimination [5–7].

Elimination of *Plasmodium vivax* malaria, the second most prevalent human malaria species in the region, remains a challenge due to the existence of a liver stage (hypnozoites) which cause further episodes (relapses) in infected subjects without the need of a new infected mosquito bite. Relapses are responsible for the vast majority of the clinical vivax episodes (up to 90%) in low transmission settings [8–10] and have been shown to cause substantial morbidity and mortality when not treated [11]. To date, the only licensed drugs available for the elimination of hypnozoites are the 8-aminoquinolines primaquine and tafenoquine. This class of drugs is associated with a dose-dependent haemolytic toxicity in individuals with glucose-6-phosphate dehydrogenase (G6PD) deficiency, an X-linked disorder of the red blood cells common in malaria endemic countries [12]. G6PD is the first enzyme of the pentose phosphate pathway and it is essential for maintaining redox equilibrium in red blood cells. G6PD deficiency can be caused by several mutations on the G6PD gene and the partial loss of enzymatic activity (i.e. deficiency) is associated with decreased capability of red blood cells to respond to oxidative stress (e.g. drug treatment, fever and ingestion of fava beans [13]). This enzymopathy is common in populations living in tropical and sub-tropical regions with an estimated global male prevalence of 8% that reaches over 20% in some populations [14]. Since the gene is located on the X-chromosome, the deficiency is found more commonly in males (in whom only one mutation is needed for the phenotype to be expressed) compared to females. In women, G6PD deficiency is expressed in those with homozygous mutations and to a lesser extent in heterozygous individuals. In this latter group, due to early X-chromosome inactivation [15], the enzymatic activity cannot be predicted by the genotype as it can be within a wide range of activity (from deficient to normal) [16]. Current phenotypic tests for detecting G6PD deficiency include quantitative laboratory-based assays such as the gold standard spectrophotometric assay [17] and the flow-cytometric assay, [18] and qualitative rapid tests such as the fluorescence spot test (FST [19]) and lateral flow tests, such as CareStart [20] and Binax Now [21]. Quantitative point-of-care tests are currently under development and validation [22].

While the single low dose (0.25 mg base/kg) primaquine used for interrupting transmission of *P. falciparum* has been showed to be safe even in G6PD deficient subjects [23], the 14-day primaquine regimen (0.25–0.5 mg base/kg daily) and single 100 mg dose of tafenoquine needed for the radical cure of *P. vivax* cannot be given to G6PD deficient patients. Reports show that even women who test normal with the qualitative test are at risk of drug-induced haemolysis when taking primaquine [9] and tafenoquine cannot be given to subjects with < 70% normal G6PD activity [24]. At present, G6PD deficient patients with *P. vivax* can be treated with a weekly dose of 0.75 mg base/kg primaquine for 8 weeks (WHO guidelines) but novel alternative regimens are under study [25].

In this context, the main objective of this study was to assess the prevalence of G6PD deficiency and to identify the most common variants causing G6PD deficiency in populations living in the Greater Mekong Subregion (GMS) where vivax malaria is frequent. Such data could support the work of policy makers in devising evidence-based strategies for the safe introduction of 8-aminoquinolines in routine malaria case management. G6PD variants were characterized in samples collected from Cambodia, Lao PDR, Myanmar, Thailand and Vietnam during National Malaria Program surveys (NMS) and population surveys (PS). Sample collection was carried out by the research units in collaboration with the National Malaria Programmes in each country. Some of the data collected in Myanmar have been already published [23]. The sample analysis was coordinated as part of a project funded by the 5% Initiative which was supported by the French Government.

## Methods

### Research partners

The study was coordinated by the Shoklo Malaria Research Unit, Faculty of Tropical Medicine, Mahidol University in Mae Sot (Thailand). Other consortium members and partners included in Cambodia, the Institut Pasteur and the National Centre for Parasitology, Entomology and Malaria Control (CNM); in Lao PDR, the Lao-Oxford-Mahosot Hospital-Wellcome Research Unit (LOMWRU) in Vientiane, the Centre of Malariology Parasitology and Entomology in Vientiane and the Institut de Recherche pour le Développement (IRD); in Thailand, the Bureau of Vector Born Diseases in Bangkok and in Vietnam, the Oxford University Clinical Research Unit in Ho Chi Minh City (OUCRU) and the Institute of Malariology, Parasitology and Entomology—Ho Chi Minh City (IMPE-HCM).

### Survey design and sample collection

#### Cambodia

The 2013 NMS was carried out from October to November 2013 during the peak malaria transmission season by a partnership of organizations supporting the CNM. This large-scale household, drug and net outlet and health facility (HF) survey was funded by the Global Fund to Fight AIDS, Tuberculosis and Malaria (Global Fund) single-stream funding (SSF). The 2013 NMS was a stratified, multistage cluster sampling design study. The country was stratified into two domains based on the malaria prevalence results of the 2007 NMS. Domain 1 consisted of the central and western provinces of Banteay Meanchey, Battambang, Kampong Speu, Kampot, Kot Kong, Oddar Meanchey, Pailin, Preah Vihear, Pursat, and Siem Reap and Domain 2 consisted of the eastern and southern provinces of Kampong Cham, Kampong Chhnang, Kampong Thom, Kratie, Mondulkiri, Rattanakiri, Sihanoukville, Takeo and Stung Treng. The survey was not nationwide as malaria transmission intensity is heterogeneous. The survey focused on populations at highest risk by stratifying first higher risk provinces and then by distance from forest. Each domain was further stratified into risk zones based on the distance from the nearest forest margin. Risk zones 1, 2, 3, and 4 correspond to distances from the forest of 0–250 m, 250–1000 m, 1–2 km, and 2–5 km, respectively. A total of 3280 households were visited and interviewed (40 households in 82 clusters; 42 clusters from Domain 1 and 40 clusters from Domain 2). Excluding households sampled in risk category 4, a total of 2726 households were sampled in villages less than two kilometres from the forest (risk categories 1–3). Within each household, a fingerprick blood sample was taken from four individuals, one from each of the following groups: one aged 0–4 years, one aged 5–14 years, one adult female, and one adult male. If there was more than one person in any of these groups, one was sampled randomly from all individuals falling in that group. In addition, any pregnant woman in the household who was not already sampled was included in the blood sampling. All study subjects gave informed consent; the study was approved by the National Ethics Committee for Health Research of the Cambodian Ministry of Health (IRB 0056 NECHR, April 23, 2013) and was conducted in compliance with the international standards for the protection of human research subjects. The current analysis has included samples from 84 villages in 64 districts and 21 provinces.

#### Lao PDR

Samples from Lao PDR were collected from two separate PS. The first one was a stratified random survey of G6PD deficiency in southern Sekong Province between September 4–26th 2014. Villages were selected by the Sekong Provincial Health Department, based on the high variety of minority groups. Everybody in the selected villages was invited to join the study. A total of 1897 written consenting villagers were recruited from six villages of two districts (Lamam and Thateng, including three villages from each district). Blood samples were collected from finger pricks and tested for malaria using rapid diagnostic tests (RDT, SD BIOLINE Malaria Ag P.f/P.v, Standard Diagnostics INC, Korea) and for G6PD phenotypic deficiency using the Trinity FST (Trinity Biotech). Participants with positive malaria RDTs were treated with artemether-lumefantrine (Coartem™) for 3 days as recommended in the Lao National Malaria Treatment Guideline and those who had G6PD phenotypic deficiency were given advice and a special Lao language card indicating their G6PD status. Three blood spots were collected onto filter papers for subsequent G6PD genotyping. The second population survey was part of the malaria Program “PALULAO, FEI No. 13INI144” which aimed to assess the prevalence of asymptomatic and submicroscopic malarial infections in the general population, including pregnant women, adults and children [26]. A cross-sectional survey was carried out in 30 randomly selected villages out of a total of the 53 villages of Vapi District (southern Salavan Province) between July and October 2014. An exhaustive list of the households and a list of the inhabitants of household by age and by sex were available with the head of village. Twenty households were randomly selected and, in each household, one adult (≥ 18 years old) and one child (between 2 and 12 years old) were randomly selected. In the same villages a contemporary survey was carried out to include all pregnant women. Peripheral blood samples were taken among a total of 1120 consenting participants for malaria using RDTs and G6PD phenotypic deficiency tests. Additional blood spots were collected onto filter papers and dried and stored at room temperature for subsequent G6PD genotyping. All surveys were approved by the National Ethics Committee for Health Research (NECHR) of Ministry of Health, Lao PDR.

#### Myanmar

In Myanmar the population survey was carried out as already published in [23]. Briefly cross-sectional surveys were conducted starting in 2013 every 3 months in four villages from two districts along the Myanmar-Thailand border in Karen state during 24 months. G6PD deficiency was determined by FST at recruitment. The protocol was reviewed and approved by the Ethics Committee of Oxford University (OxTREC no. 1017–13 and 1015–13).

#### Thailand

The NMS was carried out as a cross-sectional household survey using a stratified multi-stage cluster sampling approach where “cluster” was defined as “village” (http://www.malariaconsortium.org/media-downloads/362/Thailand%20Malaria%20Survey%202012). Samples from 5 villages in Tak province and 3 villages in Chantaburi province were analysed in this study.

#### Vietnam

In Vietnam, the population survey was carried out as described in previous study [27]. Cross sectional surveys were conducted in 6 villages located in Binh Phuoc and Ninh Thuan provinces. All villagers aged > 6 months were invited to participate in the surveys, which included the collection of venous blood samples for submicroscopic malaria detection and G6PD testing. G6PD deficiency was determined by FST at recruitment. The G6PD study protocol was reviewed and approved by the EC of Hospital for Tropical Diseases in HCMC (No.: 181/BVBND-KHTH).

Results from the current study were shared with CNM and the other partners during meetings.

### Laboratory methods

Samples collected during PS were analysed for G6PD phenotype using the FST (R&D Diagnostic, Greece) following manufacturer’s instructions. In Myanmar, the FST was performed as qualitative test with only binary deficient/normal results allowed. In Lao PDR and Vietnam, the FST was performed as semi-quantitative test thus allowing intermediate results to be reported. Blood was collected onto filter paper and DNA extracted using DNA Blood mini kits (Qiagen, Germany) in Vietnam and Cambodia and the Saponin-Chelex method [28] in Thailand and Lao PDR. Venous whole blood was collected and DNA was extracted using Blood mini kits (Favorgen Biotech Corp, Taiwan) in samples from Myanmar.

Genotyping was performed in different ways according to site and method of sampling; analysed mutations were chosen among those already described in the area. Samples from NMS were genotyped by PCR–RFLP for two common mutations (Mahidol and Viangchan) in Thailand and by PCR-based ligase detection reaction-fluorescent microsphere assay (PCR-LDR-FMA) for a panel of the 7 most frequent mutations previously described (Mahidol, Mediterranean, Coimbra, Viangchan, Chinese-5, Union and Canton) in all male samples in Cambodia [29]. Samples from PS tested with the FST and detected as G6PD deficient or G6PD intermediate were analysed by PCR–RFLP for a panel of four mutations (Viangchan, Union, Canton and Kaiping) in Vietnam and for a panel of six mutations (Viangchan, Mahidol, Chinese-4, Union, Canton, Kaiping) in Lao PDR. In Myanmar all deficient males and all women were analysed by PCR–RFLP for the same six mutations panel analysed in Lao PDR (Viangchan, Mahidol, Chinese-4, Union, Canton, Kaiping).

#### PCR–RFLP

A summary of primers and PCR conditions used is reported in Additional file 1: Table S2. Some protocols were already published [30] while others were set-up for the study. All protocols were shared between sites.

#### PCR-LDR-FMA (PCR-based ligase detection reaction-fluorescent microsphere assay)

PCR-LDR-FMA was designed to detect the seven most frequent Cambodian G6PD variants. Three PCR amplifications covering six exonic regions (Exons 6–7, 9 and 10–12) containing SNPs involved in seven G6PD variants (G6PD-Mahidol, Mediterranean, Coimbra, Viangchan, Chinese-5, Union and Canton) were carried out. Nucleotidic sequences of the PCR primers, locus-specific and allele-specific probes are provided in Additional file 2: Table S3 and extensive methods in Additional file 3. PCR and LDR reactions were conducted in 96-well plate. The fluorescence of each allele-specific LDR products was measured on a MagPix instrument with xPonent 4.2 software (LUMINEX) using the same approach as Dwivedi et al. [31].

### Maps

GPS coordinates were available for each village/district where samples were collected and analysed. Prevalence of deficiency in males (either by phenotypic test or by genotyping) was calculated at the village or district level and at the province level. Allelic frequencies of Viangchan and Mahidol variants were calculated among males at the village or district level and at the province level. The maps were made in R using the leaflet package [32]. OpenStreetMap was used for the tile base layer. The first administrative units for each country were used and GPS coordinates of sampling sites were assigned to the administrative area they fall into.

### Data analysis

Data were collected by each site independently and a pooled dataset was built in SPSS (v23) containing sample ID, gender, FST phenotype (when available), genotype (when available), village/district/province name and GPS reference (when available). Prevalence of deficiency in male was calculated from the number of observed G6PD mutated or deficient males (n) over the total number of analysed males (N), q = (n/N), standard errors (SE) were calculated as the square root of (pq/N). Since in two sites (Vietnam and Laos) only phenotypically deficient and intermediate samples were genotyped (and the proportion of heterozygous women with normal G6PD phenotype was therefore not identified) allelic frequencies of Mahidol and Viangchan mutations were calculated using data from males only. Expected genotypic frequencies in females at the country level were calculated by Hardy–Weinberg equilibrium whereby the allelic frequency of the of wild type allele is p and that of the mutation is q = (1 − p) and heterozygous women have genotypic frequency of 2pq. Data were analysed at the country level, and village or district level. In Cambodia, where the number of samples collected and analysed in each village was consistently under 25, the lower administrative level used for all analysis was the district level. The full database is available in the Additional file 4.

## Results

**Fig. 1:**
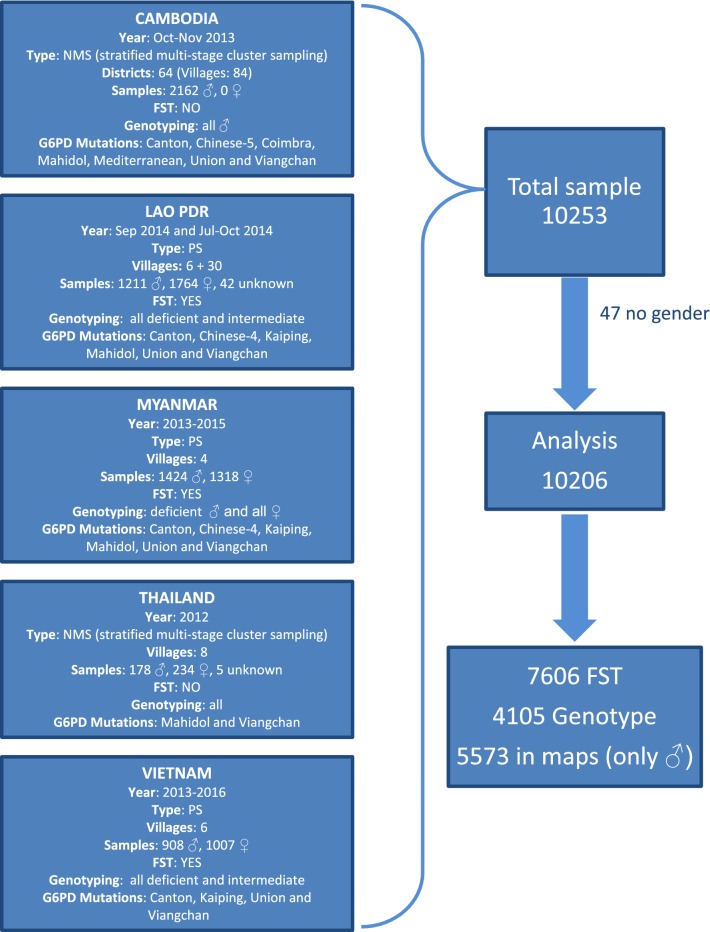
Flowchart of sample collection and analysis

Overall 10,253 samples collected in 118 villages or districts across 27 provinces in the GMS either from NMS or PS were analysed (Fig. 1 and Additional file 5: Table S1). A total of 47 samples did not have information on gender and were, therefore, excluded from analysis.

In Cambodia, 2162 samples (100% from males) in 64 districts were genotyped. In Lao PDR, 3017 samples (40.1% from males) in 36 villages were collected and analysed during PS. In Myanmar, 2742 samples (51.9% from males) were collected in 4 different villages. In Thailand, 417 samples (42.7% from males) in 8 different villages were selected among those collected during the NMS. In Vietnam 1915 samples (47.4% from males) were collected in 6 villages during population surveys. Number of samples analysed for either phenotype or genotype in each single village or district ranged from 5 to 1888 (Additional file 5: Table S1).

Out of 415 samples (310 from males and 105 from females) that lacked GPS coordinates, 261 had available G6PD phenotype and/or genotype and were therefore included in Tables 1 and 2 but not in the maps. A total of 5573 samples collected from males were used to produce the maps (Fig. 1).

**Table 1:** Results of the fluorescent spot test (FST) by gender performed with samples collected from Lao PDR, Myanmar and Vietnam

FST results	Lao PDR		Myanmar		Vietnam		Total	
	Males (%)	Females (%)	Males (%)	Females (%)	Males (%)	Females (%)	Males (%)	Females (%)
Deficient	71 (5.9)	21 (1.2)	220 (15.6)	69 (5.3)	78 (8.6)	47 (4.7)	369 (10.4)	137 (3.4)
Intermediate	35 (2.9)	58 (3.3)	0 (0.0)	0 (0.0)	51 (5.6)	67 (6.7)	86 (2.4)	125 (3.1)
Normal	1105 (91.2)	1685 (95.5)	1194 (84.4)	1233 (94.7)	779 (85.9)	893 (88.6)	3078 (87.1)	3811 (93.6)
Total	1211	1764	1414	1302	908	1007	3533	4073

**Table 2:** Distribution of the G6PD variants by gender and genotype in samples collected from Cambodia, Lao PDR, Myanmar, Thailand and Vietnam

Country	Gender	Normal^a^	Genotype	G6PD variants										% Carriers	Cumulative allelic frequency
				Total	Canton	Chinese-4	Chinese-5	Coimbra	Kaiping	Mahidol	Med	Union	Viangchan		
Cambodia^b^	Males	1756	Hemizygote	406	4	NI	2	3	NI	8	3	3	383	18.8	18.8
	Females	0	Homozygote	NI	NI	NI	NI	NI	NI	NI	NI	NI	NI	NI	NI
			Heterozygote	NI	NI	NI	NI	NI	NI	NI	NI	NI	NI	NI	NI
Lao PDR^b^	Males	1123	Hemizygote	88	1	5	NI	NI	3	2	NI	6	71	7.3	7.3
	Females	1709	Homozygote	9	–	–	NI	NI	–	–	NI	–	9	0.5	1.7^c^
			Heterozygote	42	–	1	NI	NI	1	2	NI	3	35	2.4	
Thailand	Males	165	Hemizygote	13	NI	NI	NI	NI	NI	6	NI	NI	7	7.3	7.3
	Females	188	Homozygote	3	NI	NI	NI	NI	NI	2	NI	NI	1	1.3	10.5
			Heterozygote	43	–	NI	NI	NI	NI	21	NI	NI	22	18.4	
Myanmar	Males	1222	Hemizygote	202	6	5	NI	NI	2	188	NI	–	1	14.2	14.2
	Females	998	Homozygote	34	1	–	NI	NI	–	33	NI	–	–	2.6	13.5
			Heterozygote^d^	289	6	–	NI	NI	–	279	NI	–	4	21.9	
Vietnam	Males	856	Hemizygote	52	–	NI	NI	NI	–	NI	NI	2	50	5.7	5.7
	Females	949	Homozygote	13	–	NI	NI	NI	–	NI	NI	–	13	1.3	3.5^c^
			Heterozygote	45	–	NI	NI	NI	–	NI	NI	2	43	4.5	
Total		8966		1239	18	11	2	3	6	541	3	16	639	12.1	

NI indicates that a mutation was not investigated; a dash indicates that a mutation was not found

^a^Either by genotype as wild-type by PCR–RFLP or by phenotype as normal by FST

^b^225 samples in Cambodia and 61 samples in Lao PDR are reported here with genotypes but are not represented in the maps because of missing GPS coordinates

^c^Only samples with deficient and intermediate phenotype were genotyped; therefore the allelic frequency in females is underestimated

^d^This includes five women genotyped as double heterozygous for Canton-Mahidol and two women genotyped double heterozygote for Mahidol-Viangchan. They were counted once in each mutation column

### Prevalence of G6PD deficiency

**Fig. 2:**
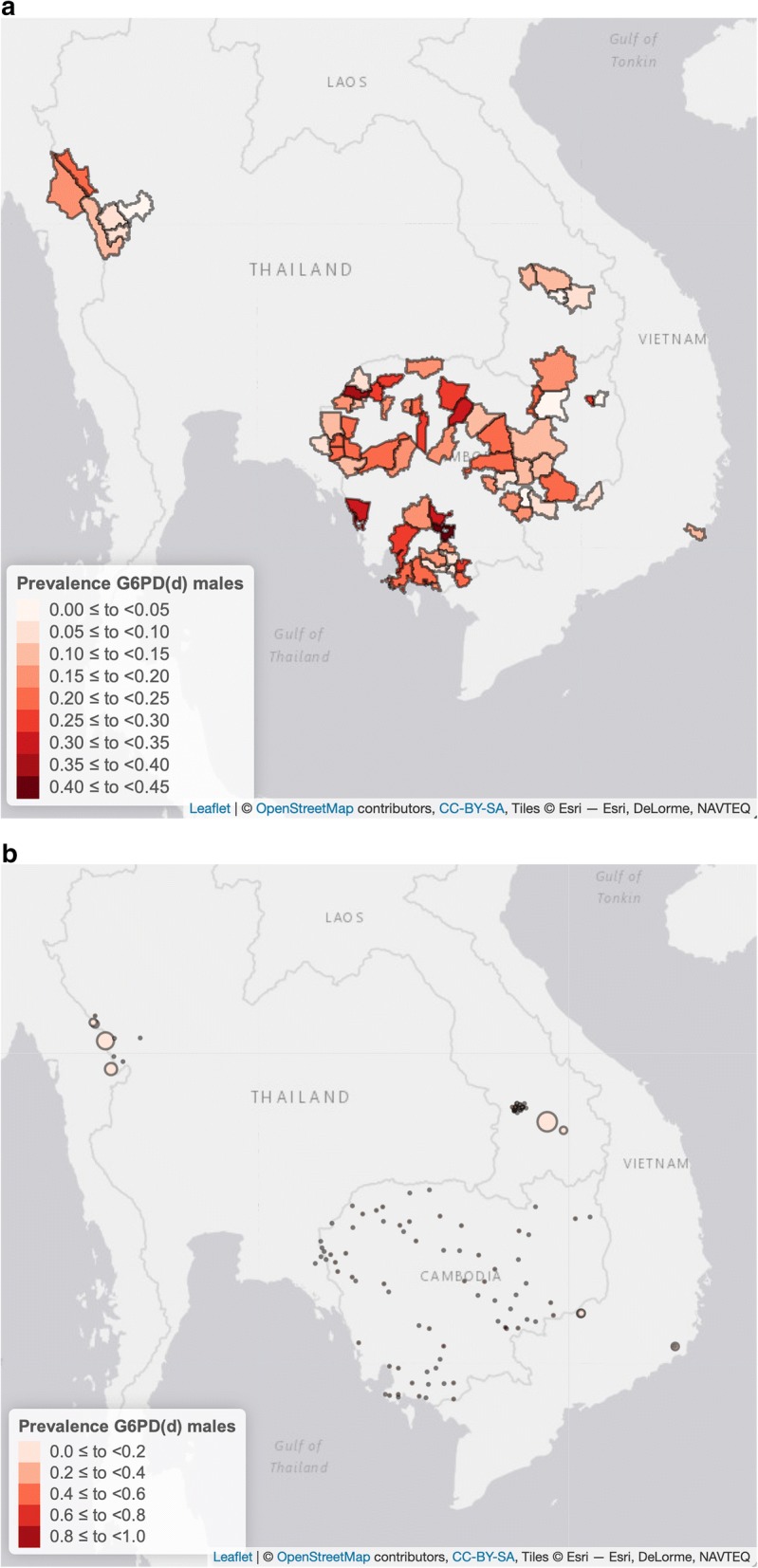
Distribution of G6PD deficiency detected in samples collected from males in the GMS at the district level (**a**) and at the village or district level (**b**). G6PD deficiency was assessed by phenotyping (FST) or genotyping (PCR–RFLP or PCR-LDR-FMA). Circles in pane **b** are proportional to sample size; for detailed prevalence data, please refer to Fig. 5 and to text

The overall prevalence of G6PD deficiency detected by the FST in samples collected from PS studies was 10.4% (369/3533) in males and 3.4% (137/4073) in females (Table 1); mean prevalence of G6PD deficiency in males was 5.9% in Lao PDR, 8.6% in Vietnam and 15.6% in Myanmar. An additional 86 males (2.4%) and 125 (3.1%) females from Lao PDR and Vietnam were found to have intermediate fluorescence. In Cambodia and Thailand, only genotyping was performed, but the frequency of males with mutations was used as a proxy for prevalence of phenotypic deficiency. Males with intermediate or normal FST results who were later genotyped and found to be carrier of a G6PD mutation were assigned a deficient phenotype for the analysis and mapping. Using only data from males, at the country level, the mean prevalence (± SE) of G6PD deficiency (tested by FST or inferred from genotype) was higher in Cambodia (18.8 ± 0.8)% and Myanmar (15.8 ± 1.0)% and lower in Vietnam (8.9 ± 1.0)%, Lao PDR (8.1 ± 0.8)% and Thailand (7.3 ± 2.0)%. As expected, a high variability was observed within each country at the provincial level (Additional file 6: Fig. S1) with prevalence of deficiency (tested by FST or inferred from genotype) ranging from 10.8 to 29.6% in Cambodia, 5.1% to 14.8% in Lao PDR, 6.7% and 8.2% in Thailand and 6.9% and 11.4% in Vietnam among different provinces. Even greater variability was observed at the district and village level (Fig. 2a) where the small sample size of some locations (< 25 subjects) did not always allow for a reliable estimation of prevalence of G6PD deficiency. At the lowest administrative levels (district in Cambodia and villages in the other countries) observed prevalence of deficiency ranged from 0.0 to 36.8% in Cambodia (with 1 village in Tbong Khumum district showing 100% prevalence based on 1 single male subject analyzed), 0.0% to 45.5% in Lao PDR, 12.9% to 17.5% in Myanmar, 0.0% to 21.4% in Thailand and 0.0% to 12.5% in Vietnam (Figs. 2b and 5a, b).

### G6PD variants across the GMS: Viangchan and Mahidol are the most prevalent and widespread

**Fig. 3:**
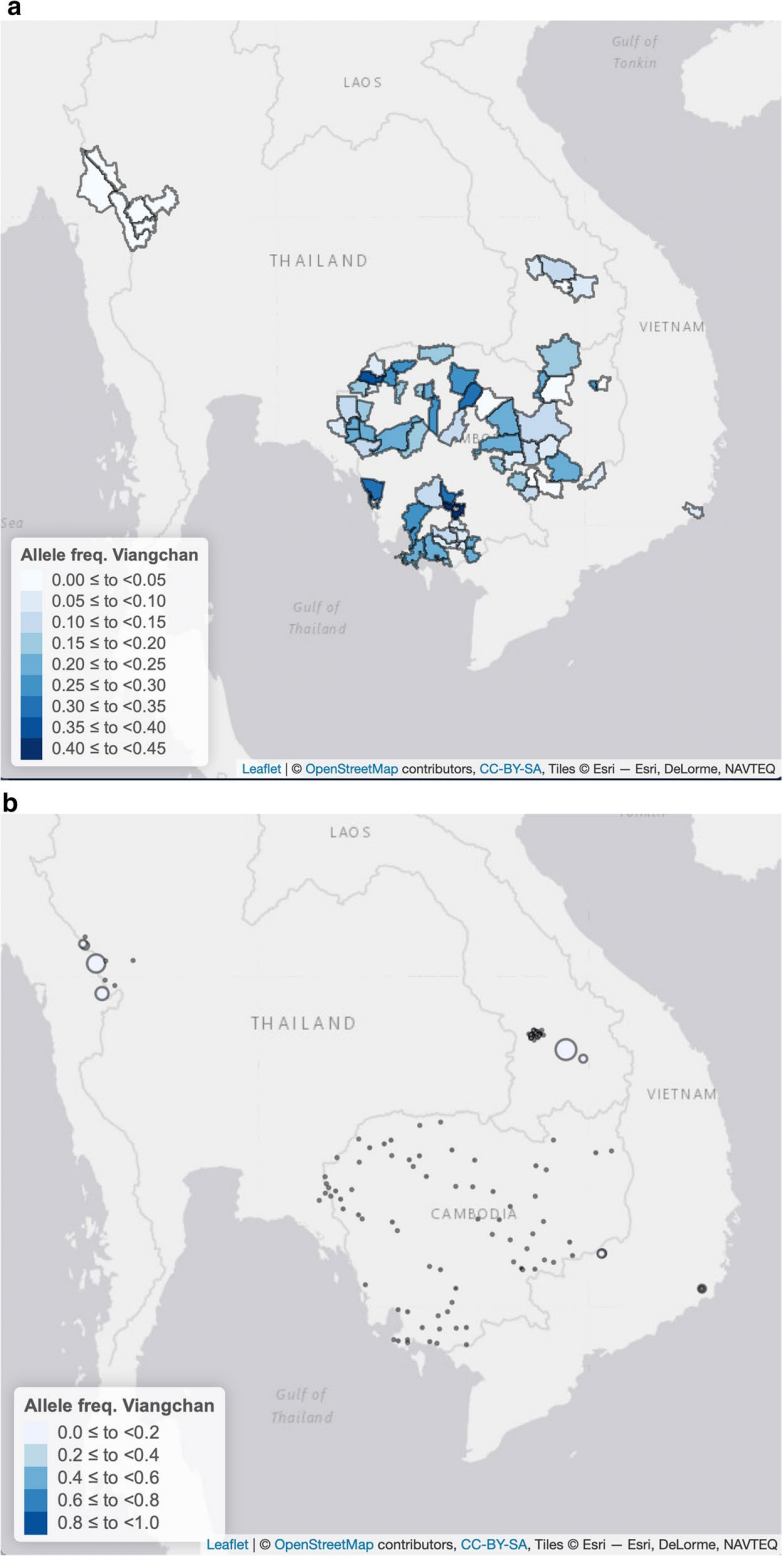
Allelic frequencies of G6PD Viangchan variant detected in samples collected from males in the GMS at the district level (**a**) and at the village or district level (**b**). Allelic frequencies were assessed by genotyping (PCR–RFLP or PCR-LDR-FMA). Circles in pane **b** are proportional to sample size

**Fig. 4:**
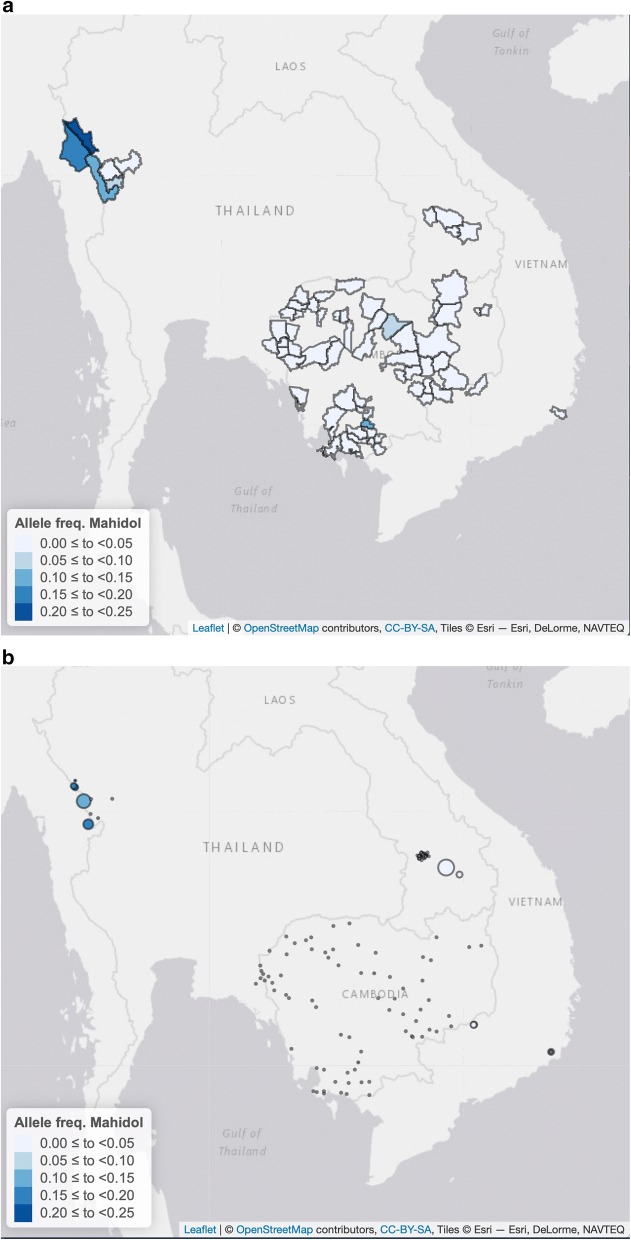
Allelic frequencies of G6PD Mahidol variant detected in samples collected from males in the GMS at the district level (**a**) and at the village or district level (**b**). Allelic frequencies were assessed by genotyping (PCR–RFLP or PCR-LDR-FMA). Circles in pane **b** are proportional to sample size

The prevalence of G6PD variants (detected by PCR–RFLP or PCR-LDR-FMA) in 10,205 samples analysed is detailed in Table 2. Allelic frequency for Viangchan and Mahidol alleles are represented in Fig. 3a, b and Additional file 7: Fig. S2 and Fig. 4a, b and Additional file 8: Fig. S3 respectively. In Lao PDR and Vietnam, only samples with deficient and intermediate phenotypes were genotyped therefore, since the allelic frequency calculated in females would not take into account heterozygous women with normal G6PD phenotype, the allelic frequencies represented in Figs. 3 and 4 were calculated only in males.

Viangchan variant was investigated in all samples and was found to be the most frequent variant among those analysed, accounting for over half of all mutations found. In samples collected from males, its allelic frequency at the country level showed a minimum of < 1% in Myanmar and was 2.3–5.7% in Thailand, 5.5% in Vietnam, 4.8–8.9% in Lao PDR up to 8.2–28.4% in Cambodia.

Mahidol variant was investigated in all sites except Vietnam and was found at a low allelic frequency in most countries, 0–0.2% in Lao PDR, 0–3.1% in Cambodia and 0.1–6.8% in Thailand, with a maximum frequency of 13.2% in Myanmar.

Union and Canton variants were investigated in all sites except Thailand; Union was found in Cambodia, Lao PDR and Vietnam while Canton variant was found in Cambodia, Lao PDR and Myanmar; Chinese-4 and Kaiping variants were only investigated in Lao PDR, Myanmar and Vietnam. Union, Canton, Kaiping and Chinese-4 mutations were found at very low frequency (< 1%) in each country.

## Discussion

Estimating the prevalence of G6PD deficiency and characterizing the underlying mutations are necessary steps to devise *P. vivax* elimination strategies in the GMS using safe 8-aminoquinolines regimens. Assessing the prevalence of G6PD deficiency by population screening with phenotypic tests such as the FST or CareStart RDT represents the ideal strategy but it is rarely done due to requirement of fresh blood samples. Genotyping on blood samples that can be batched and stored is often preferred for logistical reasons. However, in South-East Asia, populations present a relatively high molecular heterogeneity in the G6PD gene [33] and, therefore, the real prevalence of deficiency might be underestimated if only genotyping is performed. To overcome this issue, in the current study, blood samples from Lao PDR, Myanmar and Vietnam were screened first using a qualitative phenotypic test (FST) to assess the prevalence of G6PD deficiency and then genotyped (by PCR–RFLP) for those found to be fully or partially deficient to characterize the underlying mutations. In addition, in order to have a wider regional perspective on the presence and frequencies of G6PD mutations, genotyping was performed with samples already collected during NMS in Thailand and Cambodia for whom a G6PD phenotype was not available. In these two sites, published data were used to identify the most common mutations to include in genotyping analysis [23, 29, 34–38]. In particular in Cambodia, this approach allowed using a large number of already collected blood samples representative of malaria endemic areas in the country.

In the Lao PDR and Myanmar sites where the FST was carried out and a panel of six variants was investigated, a mutation could not be identified in at least 10% of samples collected from males and diagnosed as deficient by FST, 14.1% (10/71) in Lao PDR and 10.5% (23/220) in Myanmar). This proportion increased to 37.2% (29/78) in Vietnam, were four variants were studied. Although some error in the interpretation of the FST cannot be excluded, the results seem to confirm the great heterogeneity in the G6PD gene present in the investigated populations. In Cambodia, the data confirmed the heterogeneity of prevalence and spatial distribution of mutations in three exonic regions comprising the seven major mutations observed in previous studies.

Viangchan and Mahidol variants were observed at the highest prevalence in the region. In particular, Viangchan mutation tested in all sites was widely distributed in South-East Asia, with a clear western limit at the Thai-Myanmar border. The Union mutation, although much less frequent, appeared to have a similar distribution. The Mahidol mutation also showed a wide distribution, but with a steeper decline in frequency from Myanmar and Thailand to the other sites.

Mahidol and Viangchan variants have been shown to cause low or very low enzymatic activity in RBCs and are associated with relatively high hemolytic risk by 8-aminoquinolines treatment [9, 24, 39]. The other variants have not been characterized so extensively but in all the samples analysed they were associated with deficiency (in 80% of the hemizygous males) or intermediate deficiency as assessed by the FTS. When taken together, phenotypic data from PS and genotyping data from NMS in males showed that G6PD deficiency in the GMS is highly prevalent and widespread (Fig. 2b).

**Fig. 5:**
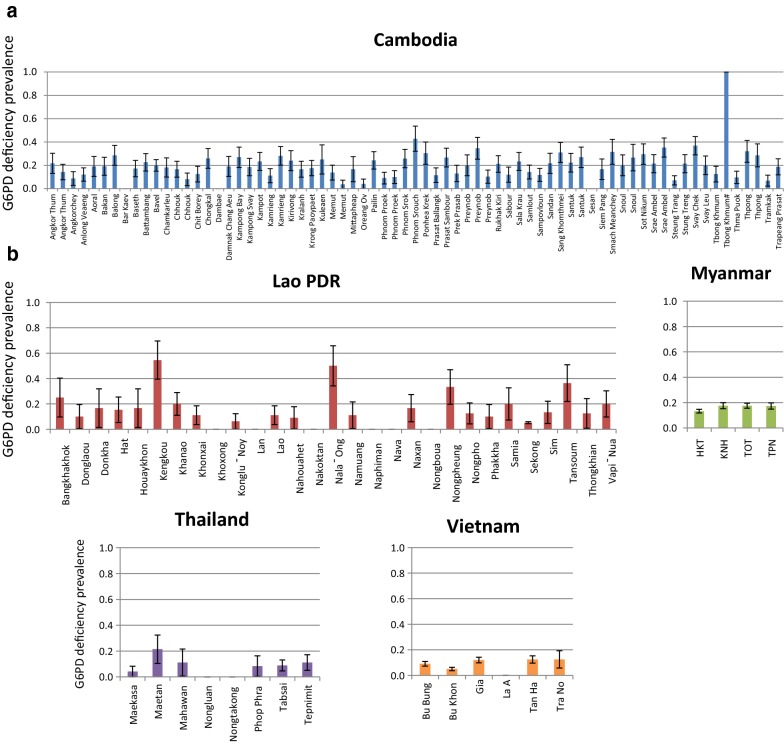
Prevalence (± SE) of G6PD deficiency detected in males at the village or district level in samples collected in Cambodia (**a**) and in Lao PDR, Myanmar, Thailand and Vietnam (**b**). On the X-axes are the names of villages or districts of each country, on the Y-axes the prevalence of G6PD deficiency. Prevalence of G6PD deficiency in Tbong Khumum# came from 1 single male

While the use of single low dose primaquine for blocking transmission of *P. falciparum* has been shown to be safe [23], the routine use of 8-aminoquinolines for *P. vivax* elimination in these large endemic areas will require G6PD testing. G6PD testing in the field will need to address in particular the challenge of identifying G6PD heterozygous women with intermediate G6PD phenotypes. By using the prevalence of G6PD deficiency detected in samples from males as a proxy for the cumulative allelic frequency of G6PD variants, it is possible to estimate the mean expected proportion of women with a heterozygous genotype in each country; in Thailand this would be on average 13.5%, in Lao PDR 14.9%, in Vietnam 16.3% and as high as 26.6% and 30.5% in Myanmar and Cambodia, respectively. Although these estimates have large confidence intervals (and with the high variability seen at the district or village levels the figures can be as low as 0% and as high as over 40%, Fig. 5a, b) they nonetheless indicate that in several villages across a vast geographical region heterozygous women represent a large proportion of women at risk of haemolysis for whom the G6PD phenotype is unpredictable. In this group of subjects, G6PD activity should be assessed with a quantitative G6PD assay before the use of radical treatment with 8-aminoquinolines, in particular tafenoquine.

Despite the large amount of data provided here, this study presents several limitations, mainly linked to the different designs of sample collection and analysis used in the different countries. First of all, although the project tried to estimate the phenotypic deficiency, the phenotypic test was not carried out in Cambodia and Thailand. Using data from the NMP surveys of these two countries was justified by the intention to test thousands of samples already collected. Second, the diagnostic performances of the FST in Lao PDR and Vietnam could not be assessed as the project resources allowed only to genotype samples with deficient and intermediate phenotype. Third, with the exception of Mahidol and Viangchan variants, the overall prevalence and distribution of the other G6PD variants could not be investigated. Last, but not least, with the exception of Cambodia, analysed samples came from a limited number of villages and districts within each region; in all study sites samples were from related subjects. Such sampling without doubt is not ideal for assessing allelic frequencies or the prevalence of G6PD deficiency, as clearly shown in the maps where the prevalence of deficiency ranged from nil up to > 40% with relatively larger SE especially in smaller collection sites. Results from few villages also make these findings less generalizable for the whole country especially in areas of great ethnic diversity such as South-East Asia. Nonetheless the overall mean prevalence of G6PD deficiency calculated here at the country level (although with large confidence intervals) remains of interest especially for policy makers and for National Malaria Programmes. The current data, together with other published evidence, show that several populations in the GMS have high prevalence of G6PD deficiency and strongly indicate that safe use of 8-aminoquinolines for vivax malaria elimination will require qualitative and quantitative G6PD testing.

## Conclusions

The results of the study confirm that G6PD deficiency is common in populations living in the Greater Mekong Subregion and is caused by several mutations. In order to eliminate vivax malaria, appropriate test-and-treatment strategies, including use of point-of-care quantitative G6PD tests, will need to be devised by the National Malaria Programmes for the safe and wide use of 8-aminoquinolines in the region.

## Additional files

**Additional file 1: Table S2.** Primers sequences, restriction enzymes and annealing temperatures of PCR-RFLP protocol used.

**Additional file 2: Table S3.** Details regarding SNP and amino acid changes of the seven main G6PD variants along with sequences of the PCR primers, allele-specific primers and locus-speficic probes used to test samples collected from Cambodian males, Cambodia, National Malaria Survey 2013.

**Additional file 3.** PCR-based ligase detection reaction-fluorescent microsphere assay (PCR-LDR-FMA) protocol.

**Additional file 4.** Study dataset.

**Additional file 5: Table S1.** Number and G6PD status of analyzed samples by village or district level.

**Additional file 6: Fig. S1.** Distribution of G6PD deficiency detected in samples collected from males in the GMS at the province level.

**Additional file 7: Fig. S2.** Allelic frequencies of G6PD Viangchan variant detected in samples collected from males in the GMS at the province level.

**Additional file 8: Fig. S3.** Allelic frequencies of G6PD Mahidol variant detected in samples collected from males in the GMS at the province level.

## Abbreviations

G6PD: glucose-6-phosphate dehydrogenase
ACT: artemisinin combination therapy
NMS: National Malaria Survey
PS: population survey
GMS: Greater Mekong Subregion
FST: fluorescent spot test
PCR: polymerase chain reaction
RFLP: restriction fragment length polymorphism
LDR-FMA: ligase detection reaction-fluorescent microsphere assay
