# Supplementary material for: Molecular characterization and mapping of glucose-6-phosphate dehydrogenase (G6PD) mutations in the Greater Mekong Subregion
Source: Malar J. 2019 Jan 23;18:20. doi: 10.1186/s12936-019-2652-y (PMC6343352; doi:10.1186/s12936-019-2652-y)
Supplement: Supplementary file 1 — Additional file 1: Table S2. Primers sequences, restriction enzymes and annealing temperatures of PCR-RFLP protocol used. [file 12936_2019_2652_MOESM1_ESM.docx]

**Table S2.** Primers sequences, restriction enzymes and annealing temperatures of PCR-RFLP protocol used

| **Assay** | **Oligonucleotides** | **T_ann_** | **Restriction enzyme** | **Reference** |
| --- | --- | --- | --- | --- |
| Mahidol | 5’-GCGTCTGAATGATGCAGCTCTGAT-3’  5’-CTCCACGATGATGCGGTTCAAGC-3’ | 58°C | *Hind* III | Huang *et al.*, 1996 |
| Viangchan | 5’-CCTGAGGGCTGCACATCT-3’  5’-GTCGTCCAGGTACCCTTTGGGG-3’ | 64°C | *Hpy* 188III | - |
| Union | 5’-ACGTGAAGCTCCCTGACGC-3’  5’-GTGAAAATACGCCAGGCCTTA-3’ | 58°C | *Hha* I | Huang *et al.*, 1996 |
| Canton | 5’-ACGTGAAGCTCCCTGACGC-3’  5’-GTGAAAATACGCCAGGCCTTA-3’ | 58°C | *Afl* II | Huang *et al.*, 1996 |
| Kaiping | 5’-ACGTGAAGCTCCCTGACGC-3’  5’-GTGCAGCAGTGGGGTGAACATA-3’ | 58°C | *Nde* I | Huang *et al.*, 1996 |
| Chinese- 4 | 5’-CTGGAGGACTTCTTTGCCCGC-3’  5’-GGAGCACTGCCTGGGCCAGC-3’ | 64°C | *Dra* III HF | - |
