# Supplementary material for: Molecular characterization and mapping of glucose-6-phosphate dehydrogenase (G6PD) mutations in the Greater Mekong Subregion
Source: Malar J. 2019 Jan 23;18:20. doi: 10.1186/s12936-019-2652-y (PMC6343352; doi:10.1186/s12936-019-2652-y)
Supplement: Supplementary file 2 — Additional file 2: Table S3. Details regarding SNP and amino acid changes of the seven main G6PD variants along with sequences of the PCR primers, allele-specific primers and locus-speficic probes used to test samples collected from Cambodian males, Cambodia, National Malaria Survey 2013. [file 12936_2019_2652_MOESM2_ESM.docx]

**Table S3.** Details regarding SNP and amino acid changes of the seven main G6PD variants along with sequences of the PCR primers, allele-specific primers and locus-speficic probes used to test samples collected from Cambodian males, Cambodia, National Malaria Survey 2013.

| **Variant** | **G6PD-Mahidol** | **G6PD-Mediterranean** |
| --- | --- | --- |
| Exon | Exon 6 | Exon 6 |
| SNP | 487G>A | 563C>T |
| Amino acid change | G163S | S188F |
| Forward primer | ACACAAGGCACGGGAGGT | ACACAAGGCACGGGAGGT |
| Reverse primer | GAGGAGCTCCCCCAAGATAG | GAGGAGCTCCCCCAAGATAG |
| Locus-specific probe | [Phos]GCTGGAACCGCATCATC[BtnTg] | [Phos]CCTGTTCCGTGAGGACCA[BtnTg] |
| Allele-specific primer (wild-type) | *acttatttcttcactactatatca*CGGGCTCCCAGCAGAG | *attaaacaactcttaactacacaa*CTGTCCAACCACATCTCCTC |
| Allele-specific primer (mutant) | *catcttcatatcaattctcttatt*CGGGCTCCCAGCAGAA | *tacaacatctcattaacatataca*CTGTCCAACCACATCTCCTT |
|  |  |  |
| **Variant** | **G6PD-Coimbra** | **G6PD-ViangChan** |
| Exon | 6 | 9 |
| SNP | 592C>T | 871G>A |
| Amino acid change | R198C | V291M |
| Forward primer | ACACAAGGCACGGGAGGT | CCTGAGGGCTGCACATCT |
| Reverse primer | GAGGAGCTCCCCCAAGATAG | GTGCGTGAGTGTCTCAGTGG |
| Locus-specific probe | [Phos]GCATCGACCACTACCTGGG[BtnTg] | [Phos]TGTTGAAATGCATCTCAGAGGT[BtnTg] |
| Allele-specific primer (wild-type) | *attcaatactatctaacacttact*CCGTGAGGACCAGATCTACC | *cactacacatttatcataacaaat*TGGCTTTCTCTCAGGTCAAGG |
| Allele-specific primer (mutant) | *acaaatatctaactactatcacaa*CCGTGAGGACCAGATCTACT | *aactttctctctctattcttattt*TGGCTTTCTCTCAGGTCAAGA |
|  |  |  |
| **Variant** | **G6PD-Chinese-5** | **G6PD-Union** |
| Exon | 9 | 11 |
| SNP | 1024C>T | 1360C>T |
| Amino acid change | L342F | R454C |
| Forward primer | CCTGAGGGCTGCACATCT | TGAGACACTCACGCACTGGT |
| Reverse primer | GTGCGTGAGTGTCTCAGTGG | TGAGGTAGCTCCACCCTCAC |
| Locus-specific probe | [Phos]TCTATGTGGAGAATGAGAGGTGG[BtnTg] | [Phos]GCAGGTGAGGCCCAGC[BtnTg] |
| Allele-specific primer (wild-type) | *aatcaacacacaataacattcata*CTTTTGCAGCCGTCGTCC | *tcatcactttctttactttacatt*GCCAGATGCACTTCGTGC |
| Allele-specific primer (mutant) | *caatttacatttcactttcttatc*CTTTTGCAGCCGTCGTCT | *tacacaatattcatcataactaac*GCCAGATGCACTTCGTGT |
| **Variant** | **G6PD-Canton** |  |
| Exon | 12 |  |
| SNP | 1376G>T |  |
| Amino acid change | R459L |  |
| Forward primer | TGAGACACTCACGCACTGGT |  |
| Reverse primer | TGAGGTAGCTCCACCCTCAC |  |
| Locus-specific probe | [Phos]GTGAGGCCTGGCGTATTTT[BtnTg] |  |
| Allele-specific primer (wild-type) | *ttaaacaatctactattcaatcac*CAGAGCGACGAGCTCCG |  |
| allele-specific primer (mutant) | *tctctttaaacacattcaacaata*CAGAGCGACGAGCTCCT |  |
