# Supplementary material for: Molecular characterization and mapping of glucose-6-phosphate dehydrogenase (G6PD) mutations in the Greater Mekong Subregion
Source: Malar J. 2019 Jan 23;18:20. doi: 10.1186/s12936-019-2652-y (PMC6343352; doi:10.1186/s12936-019-2652-y)
Supplement: Supplementary file 3 — Additional file 3. PCR-based ligase detection reaction-fluorescent microsphere assay (PCR-LDR-FMA) protocol. [file 12936_2019_2652_MOESM3_ESM.docx]

***PCR-based ligase detection reaction-fluorescent microsphere assay (PCR-LDR-FMA) protocol.***

PCR-LDR-FMA was designed to detect the seven most frequent Cambodian G6PD variants. Three PCR amplifications covering six exonic regions (Exons 6-7, 9 and 10-12) containing SNPs involved in seven G6PD variants (G6PD-Mahidol, Mediterranean, Coimbra, Viangchan, Chinese-5, Union and Canton) were carried out.

PCR were done in 55 μL of final volume with 5 μL of DNA, 0.4 μM of each corresponding primers, 0.25 mM of each deoxynucleoside triphosphate (dNTP) (Solis Biodyne), 1× of reaction Buffer, 2.5 mM of MgCl2, 2.5 U FirePol® Taq DNA Polymerase (Solis Biodyne), with the following conditions: 94 °C for 15 min, then 40 cycles of a three step program: 94 °C for 30 s, 58 °C for 90 s (Exons 6-7,) or 60 s (Exon 9, Exonx 10-12), 72 °C for 2 min (Exons 6-7) or 2 min 30 s (Exon 9, Exonx 10-12) and final extension at 72 °C for 10 min. PCR products were pooled together and one microliter was used for the ligase detection reaction (LDR). The LDR was based on two allele-specific primers (wild-type and mutant) and one locus-specific probes. The allele-specific primers were composed of two parts: the 5-prime part hybridizing with the MagPlex-Tag probe and the 3-primer part hybridizing with the PCR product. Locus-specific probes were 5′ phosphorylated and 3′ biotinylated. Ligation was performed after hybridization of the locus-specific primer. LDRs were performed in a final volume of 15 μL holding in 1× of Taq Ligase buffer, 10 nM of each LDR allele- and locus-specific primers), 4 U of Taq DNA ligase (Genesearch) and 1 μL of pooled PCR products. Thermocycling conditions were carried out by denaturation of the double stranded DNA at 95 °C for 1 min, followed by 32 cycles at 95 °C for 15 s and hybridization at 59 °C for 2 min. Samples obtained from individuals harbouring one of the seven targeted G6PD variants (some were provided by SMRU) were used as quality control.

A 5 μL fraction of the LDR product was added to 60 μL of hybridization solution TMAC buffer (3× of tetramethylammonium chloride [TMAC] (Sigma-Aldrich), 3 mM of EDTA (Gibco), 50 mM Tris–HCl, pH 8.0 (Sigma-Aldrich), 0.1 % sodium dodecyl sulfate) and 1000 beads of each MagPlex-Tag microspheres used in the multiplex LDR. Mixtures were heated to 95 °C for 90 s and incubated at 37 °C for 35 min to allow hybridization between SNP specific LDR products (Tag-probe) and bead-labeled anti-TAG probes. Then, 6 μL of 1:50 dilution of streptavidin-R-phycoerythrin (Invitrogen) in TMAC buffer was added to the post-LDR mixture and incubated at 37 °C for 20 min in 96-well plate (Eppendorf).

PCR and LDR reactions were conducted in 96-well plate. The fluorescence of each allele-specific LDR products was measured on a MagPix instrument with xPonent 4.2 software (LUMINEX) using the same approach as Dwivedi et al., 2016. SNPs were detected when signals were above the value of the ratio signal observed with negative controls. The measurement of the signal for an allele was decomposed into the signal intensity without noise and the background noise. Negative samples show reduced signal-to-noise ratio and positive samples show increased signal-to-noise ratio. The identification of negative and positive samples was based on a classification method which minimizes the variance associated to the two series of measures. This algorithm was analogous to the k-mean algorithm where k = 2. A test was used to address each measurement to the negative or positive value of the allele.
