# Supplementary figures and images for: Molecular characterization and mapping of glucose-6-phosphate dehydrogenase (G6PD) mutations in the Greater Mekong Subregion
Source: Malar J. 2019 Jan 23;18:20. doi: 10.1186/s12936-019-2652-y (PMC6343352; doi:10.1186/s12936-019-2652-y)

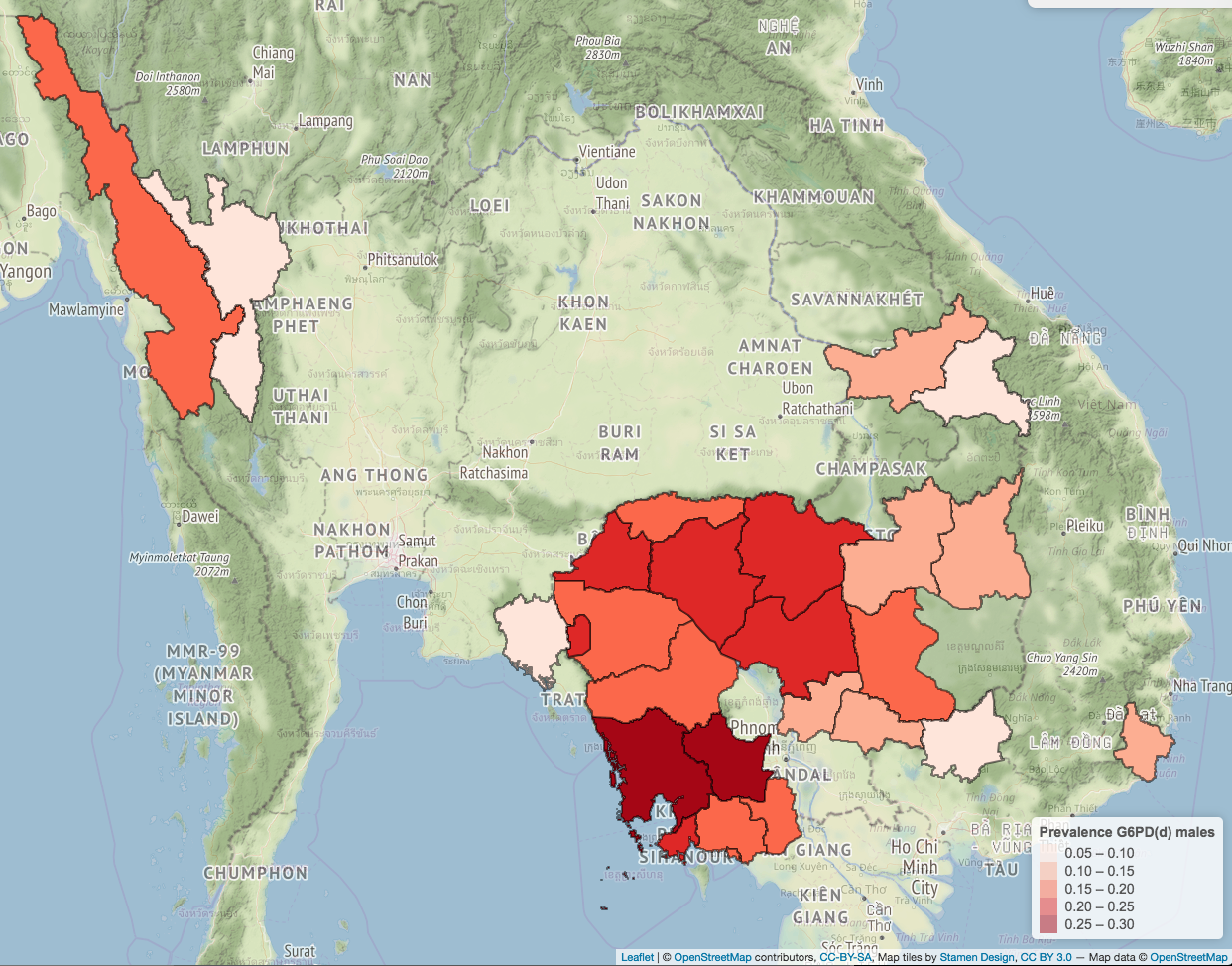

Supplement: Supplementary file 6 — Additional file 6: Fig. S1. Distribution of G6PD deficiency detected in samples collected from males in the GMS at the province level. [file 12936_2019_2652_MOESM6_ESM.png]

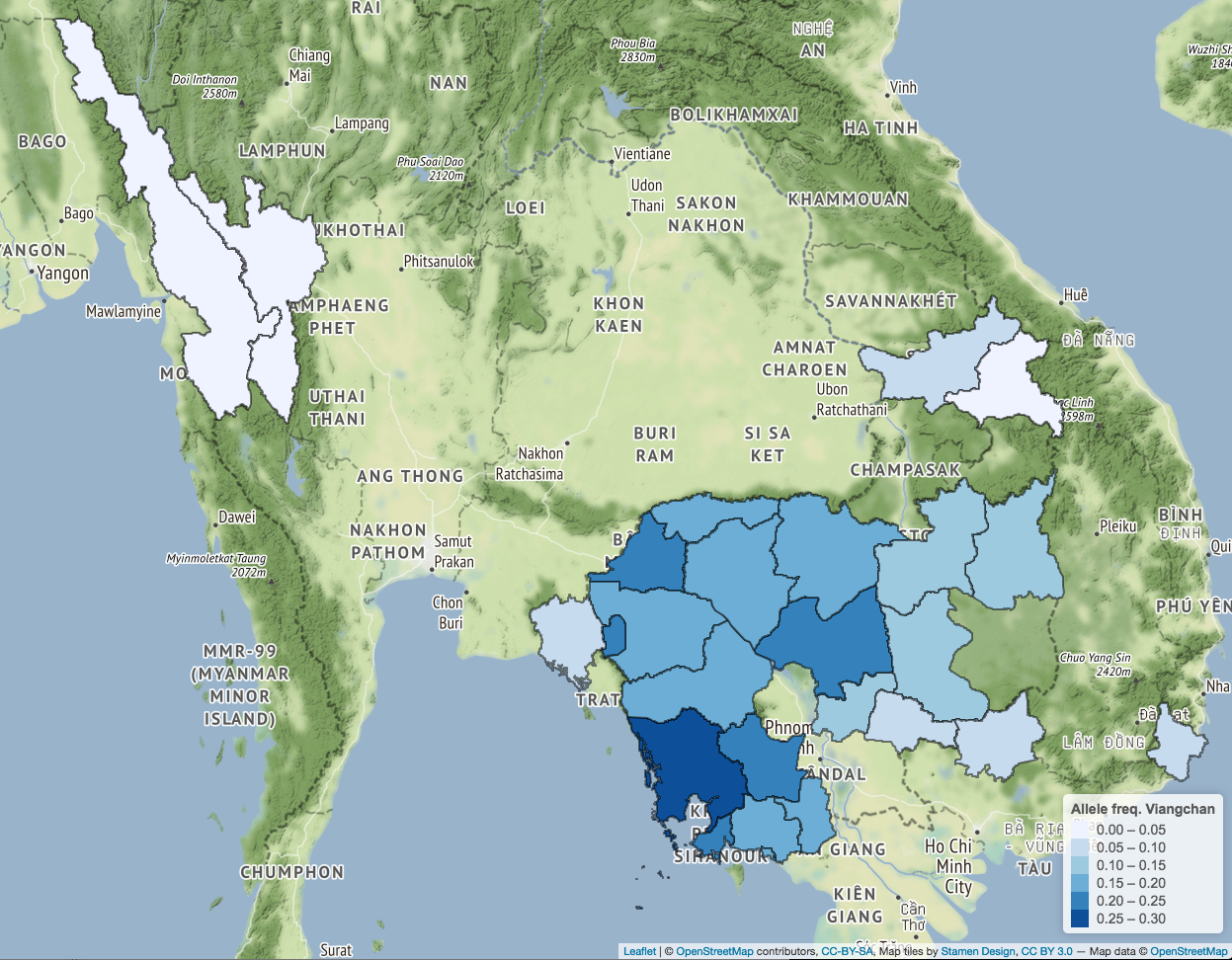

Supplement: Supplementary file 7 — Additional file 7: Fig. S2. Allelic frequencies of G6PD Viangchan variant detected in samples collected from males in the GMS at the province level. [file 12936_2019_2652_MOESM7_ESM.png]

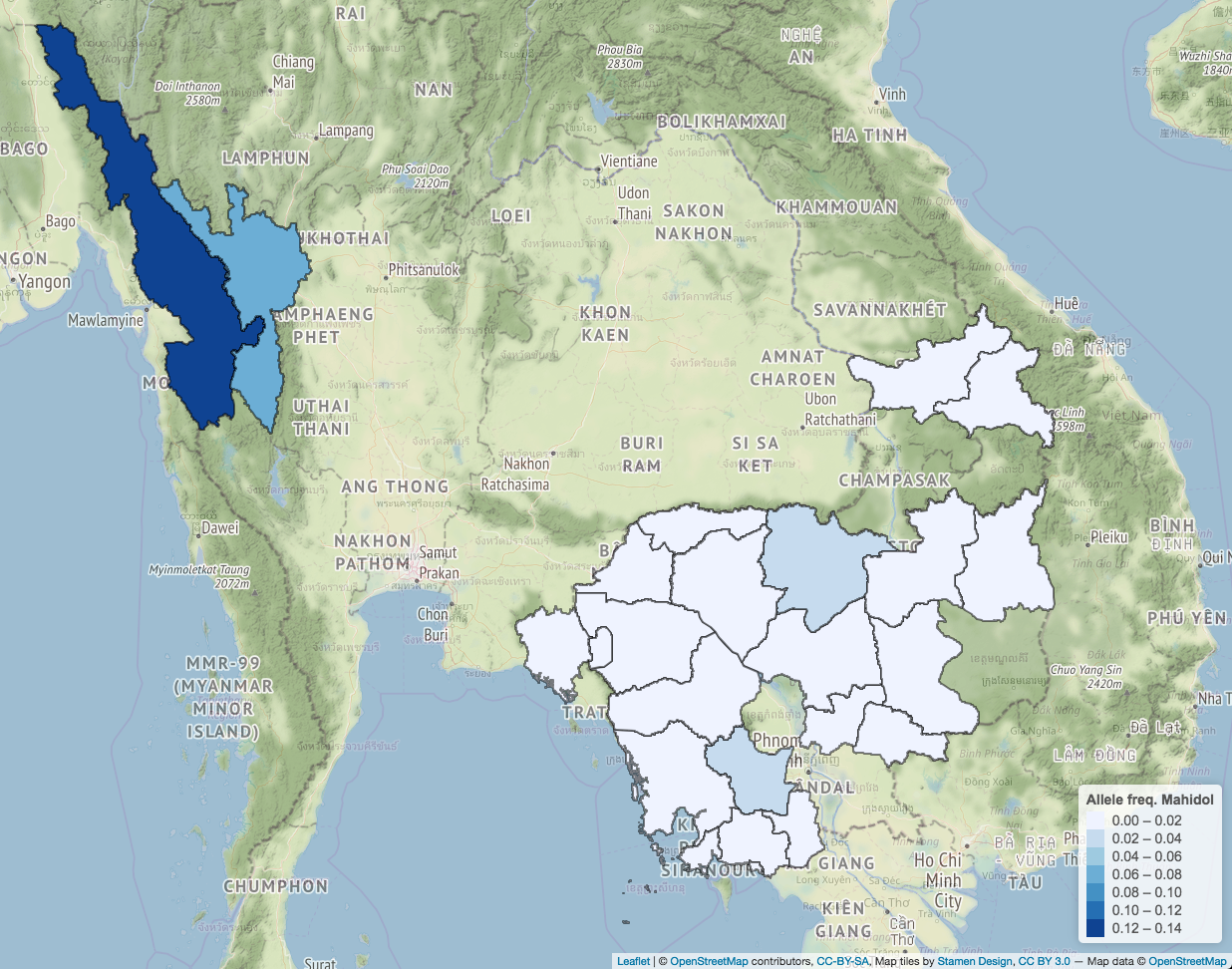

Supplement: Supplementary file 8 — Additional file 8: Fig. S3. Allelic frequencies of G6PD Mahidol variant detected in samples collected from males in the GMS at the province level. [file 12936_2019_2652_MOESM8_ESM.png]
